# Supplementary material for: Thermoplastic Starch with Poly(butylene adipate-co-terephthalate) Blends Foamed by Supercritical Carbon Dioxide
Source: Polymers (Basel). 2022 May 11;14(10):1952. doi: 10.3390/polym14101952 (PMC9145724; doi:10.3390/polym14101952)
Supplement: Supplementary file 1 [file polymers-14-01952-s001.zip › polymers-1700487-supplementary.pdf]

Supporting Information

Thermoplastic Starch with Poly(butylene adipate-co-terephthalate) Blends Foamed by Supercritical Carbon Dioxide

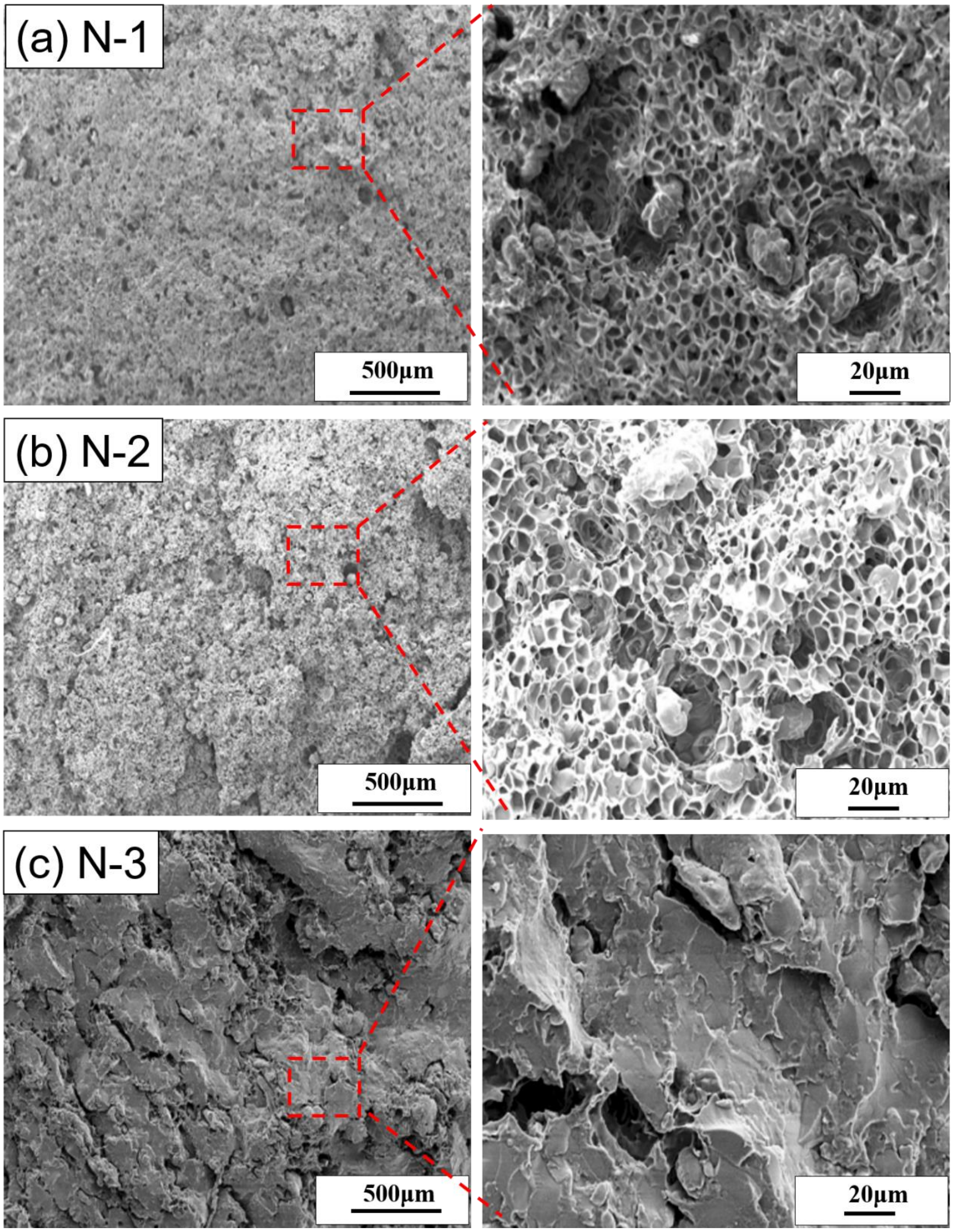

Figure S1. The SEM images of cell structure at the foaming temperature of 95 ℃ and the foaming pressure of 23.8 MPa. (a) [N-1], (b) [N-2], and (c) [N-3].

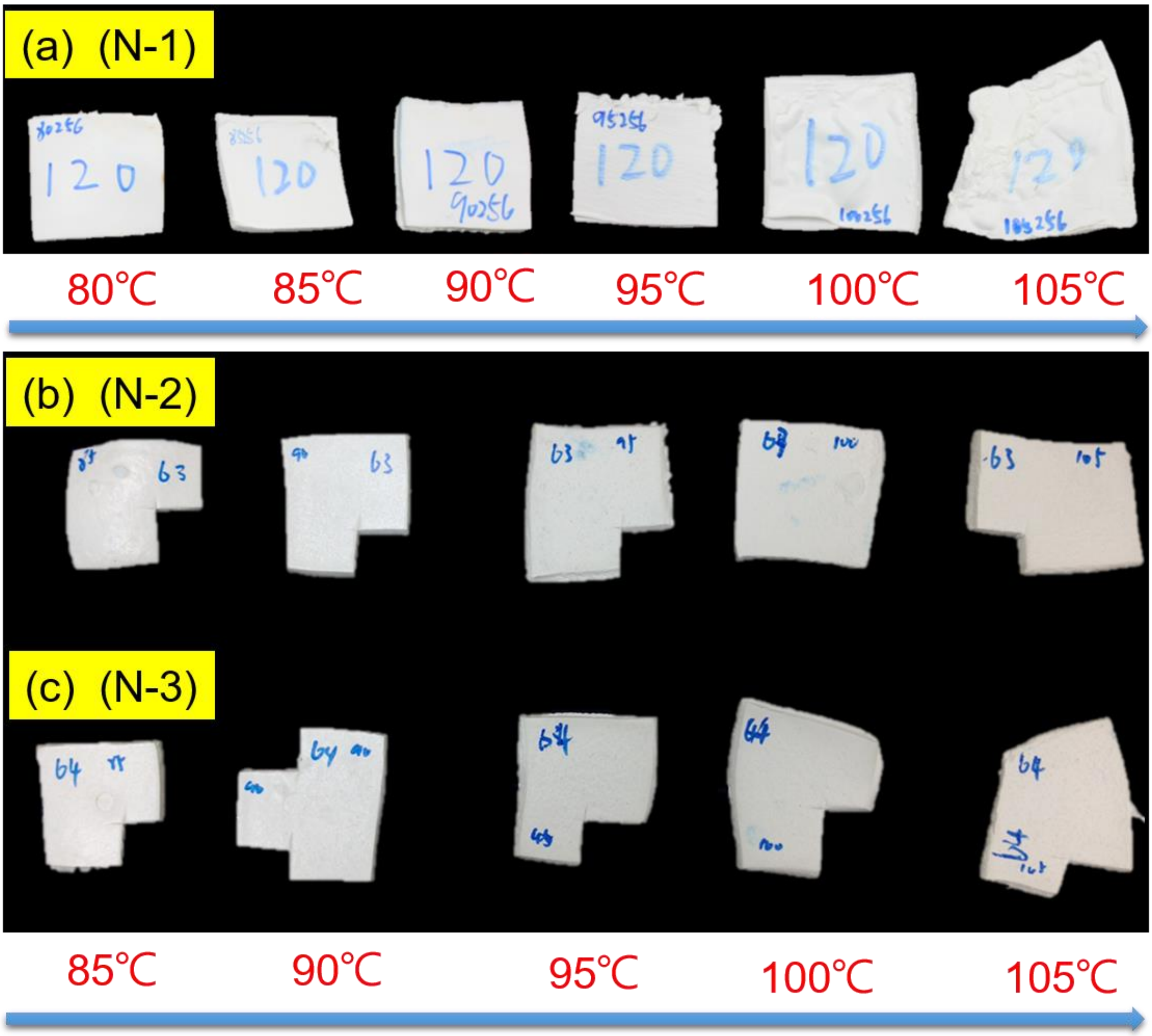

Figure S2. Dependence of TPS/PBAT foam appearance on six foaming temperatures and the foaming pressure of 17 MPa. (a) [N-1], (b) [N-2], and (c) [N-3].

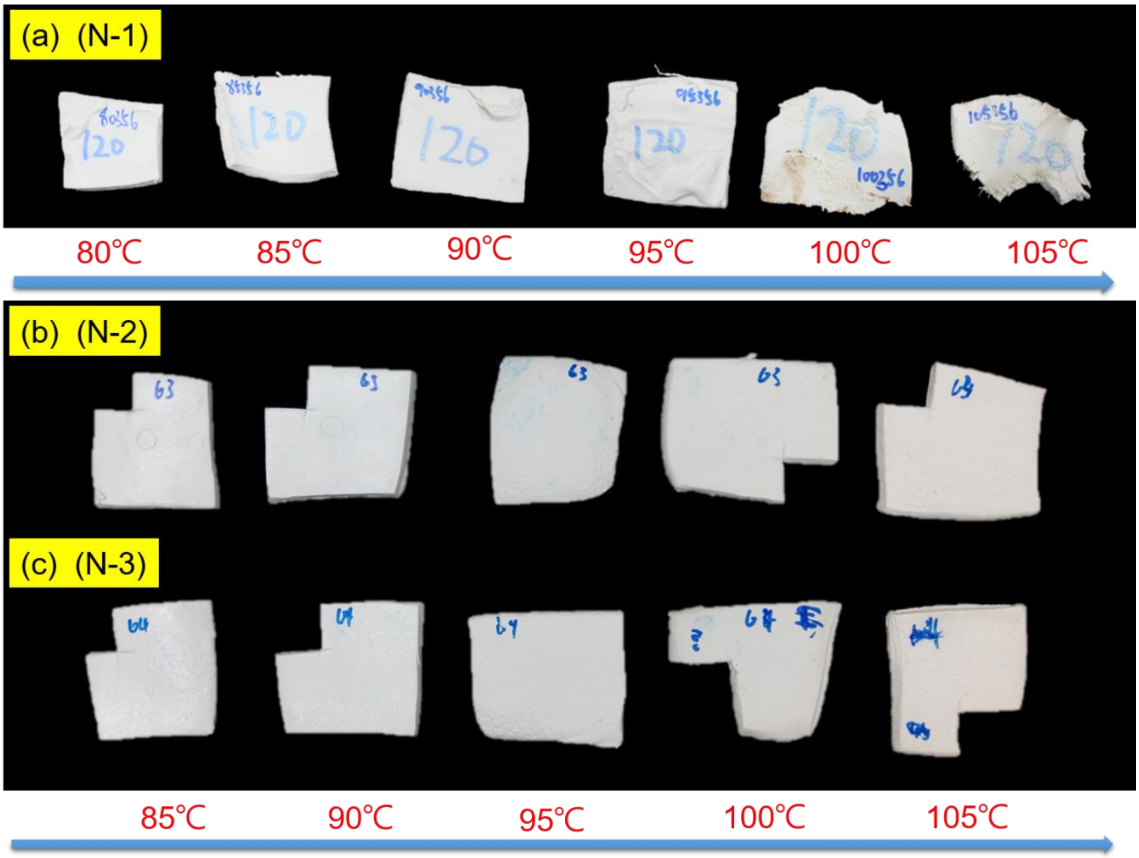

Figure S3. Dependence of TPS/PBAT foam appearance (a) [N-1], (b) [N-2], and (c) [N-3] on six foaming temperatures and the foaming pressure of 23.8 MPa.

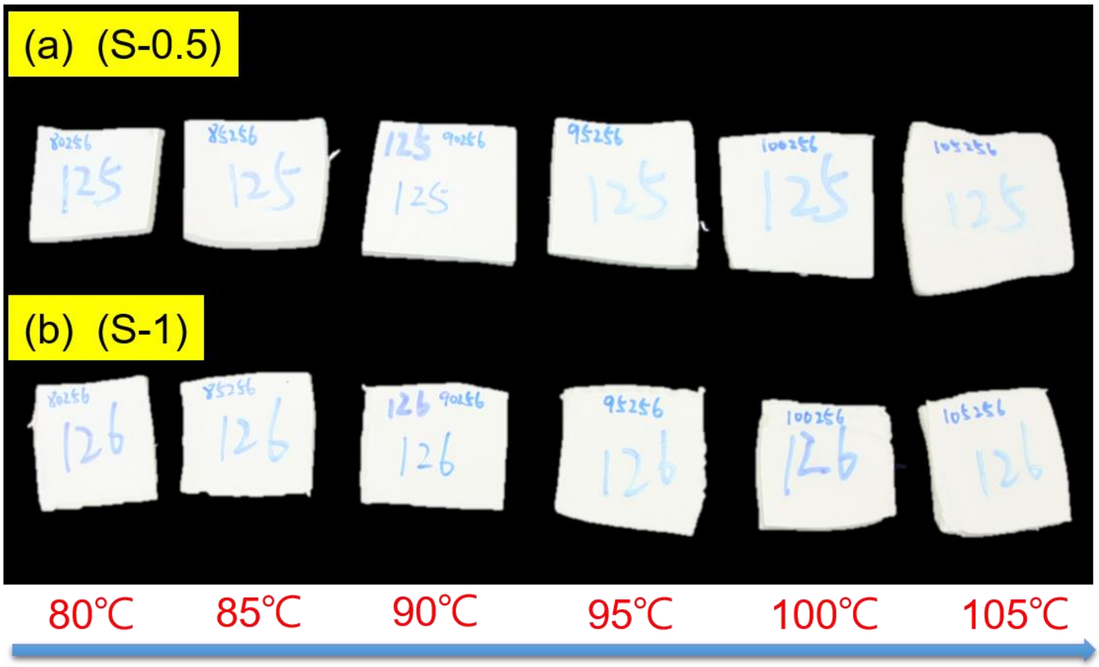

Figure S4. Dependence of (TPS with SA)/ PBAT foam appearance (a) [S-0.5] and (b) [S-1] on six foaming temperatures and the foaming pressure of 17 MPa.

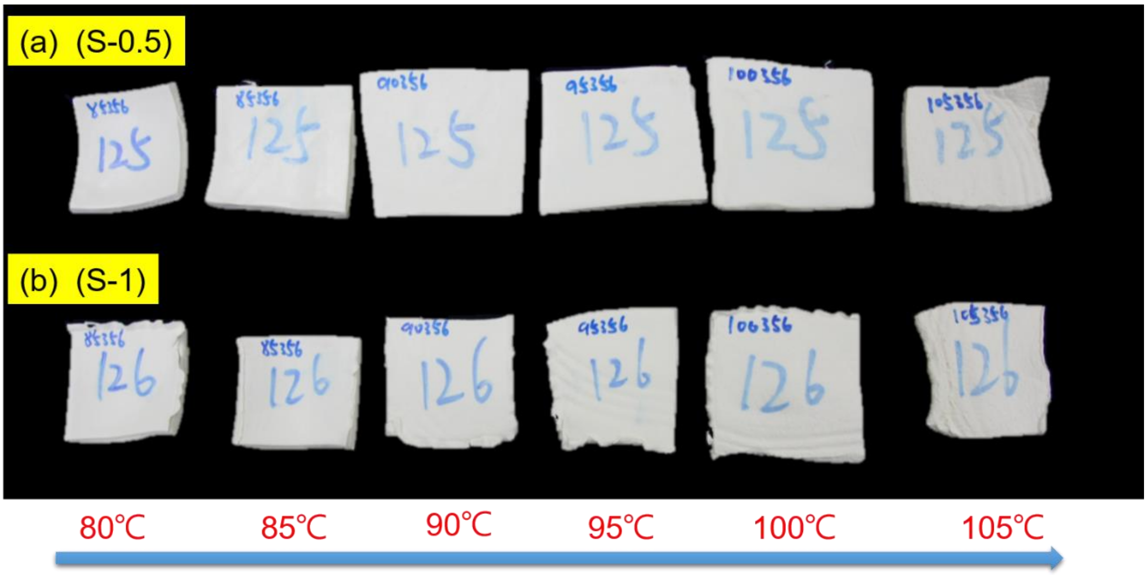

Figure S5. Dependence of TPS/PBAT foam appearance (a) [S-0.5] and (b) [S-1] on six foaming temperatures and the foaming pressure of 23.8 MPa.

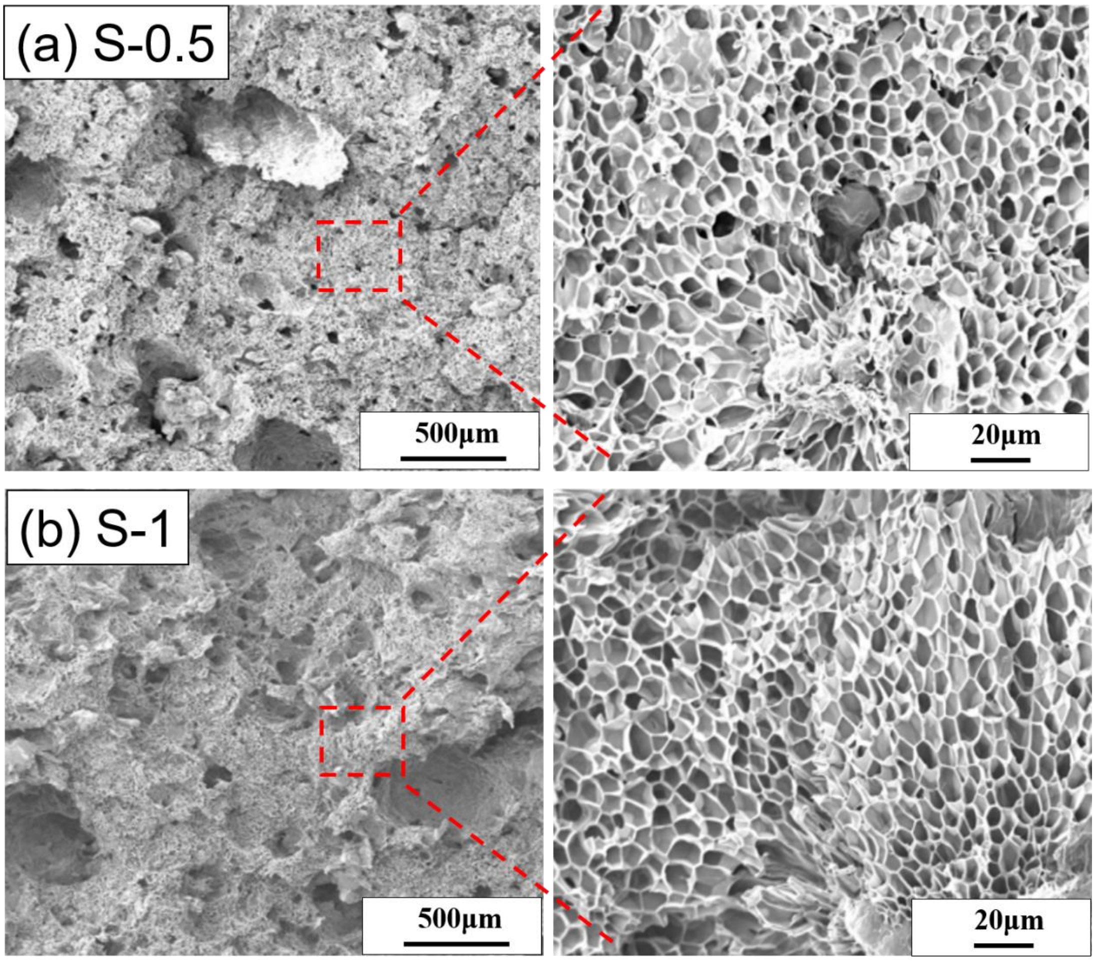

Figure S6.The SEM images of cell structure (a) [S-0.5] and (b) [S-1] at the foaming temperature of 95 ℃ and the foaming pressure of 23.8 MPa.

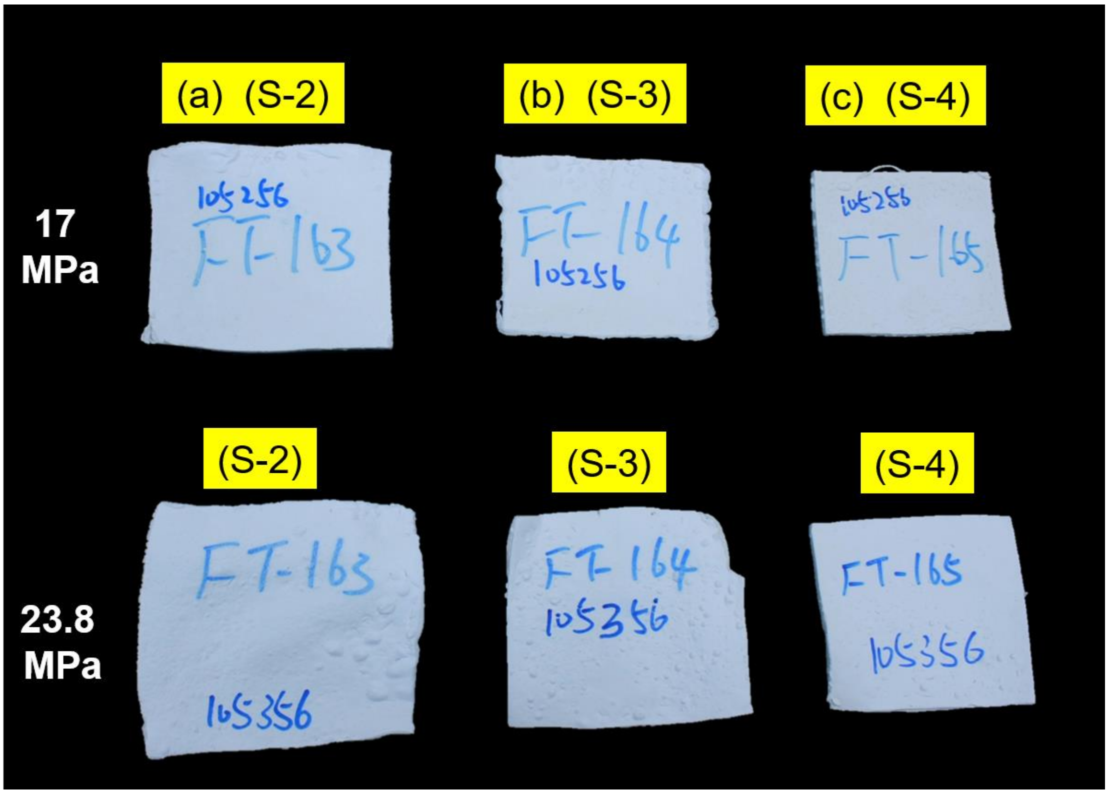

Figure S7. The appearance change of the (a) [S-2], (b) [S-3], and (c) [S-4] foam under the different foaming pressure of 17 and 23.8 MPa.
